# Efficacies and ADME properties of redox active methylene blue and phenoxazine analogues for use in new antimalarial triple drug combinations with amino-artemisinins

**DOI:** 10.3389/fphar.2023.1308400

**Published:** 2024-01-08

**Authors:** Daniel J. Watson, Lizahn Laing, Jacobus P. Petzer, Ho Ning Wong, Christopher J. Parkinson, Lubbe Wiesner, Richard K. Haynes

**Affiliations:** ^1^ Division of Clinical Pharmacology, Department of Medicine, University of Cape Town, Cape Town, South Africa; ^2^ Centre of Excellence for Pharmaceutical Sciences, Faculty of Health Sciences, North-West University, Potchefstroom, South Africa; ^3^ Rural Health Research Institute, Charles Sturt University, Orange, NSW, Australia; ^4^ School of Dentistry and Medical Sciences, Charles Sturt University, Orange, NSW, Australia

**Keywords:** malaria, artemisone, benzo[a]phenoxazine, combinations, synergism

## Abstract

Efforts to develop new artemisinin triple combination therapies effective against artemisinin-tolerant strains of *Plasmodium falciparum* based on rational combinations comprising artemisone or other amino-artemisinins, a redox active drug and a third drug with a different mode of action have now been extended to evaluation of three potential redox partners. These are the diethyl analogue AD01 of methylene blue (MB), the benzo [α]phenoxazine PhX6, and the thiosemicarbazone DpNEt. IC_50_ values *in vitro* against CQ-sensitive and resistant *P. falciparum* strains ranged from 11.9 nM for AD01–41.8 nM for PhX6. PhX6 possessed the most favourable pharmacokinetic (PK) profile: intrinsic clearance rate CL_int_ was 21.47 ± 1.76 mL/min/kg, bioavailability was 60% and half-life was 7.96 h. AD01 presented weaker, but manageable pharmacokinetic properties with a rapid CL_int_ of 74.41 ± 6.68 mL/min/kg leading to a half-life of 2.51 ± 0.07 h and bioavailability of 15%. DpNEt exhibited a half-life of 1.12 h and bioavailability of 8%, data which discourage its further examination, despite a low CL_int_ of 10.20 mL/min/kg and a high C_max_ of 6.32 µM. Efficacies of AD01 and PhX6 were enhanced synergistically when each was paired with artemisone against asexual blood stages of *P. falciparum* NF54 *in vitro*. The favourable pharmacokinetics of PhX6 indicate this is the best partner among the compounds examined thus far for artemisone. Future work will focus on extending the drug combination studies to artemiside *in vitro*, and conducting efficacy studies *in vivo* for artemisone with each of PhX6 and the related benzo[α]phenoxazine SSJ-183.

## Introduction

**FIGURE 1:**
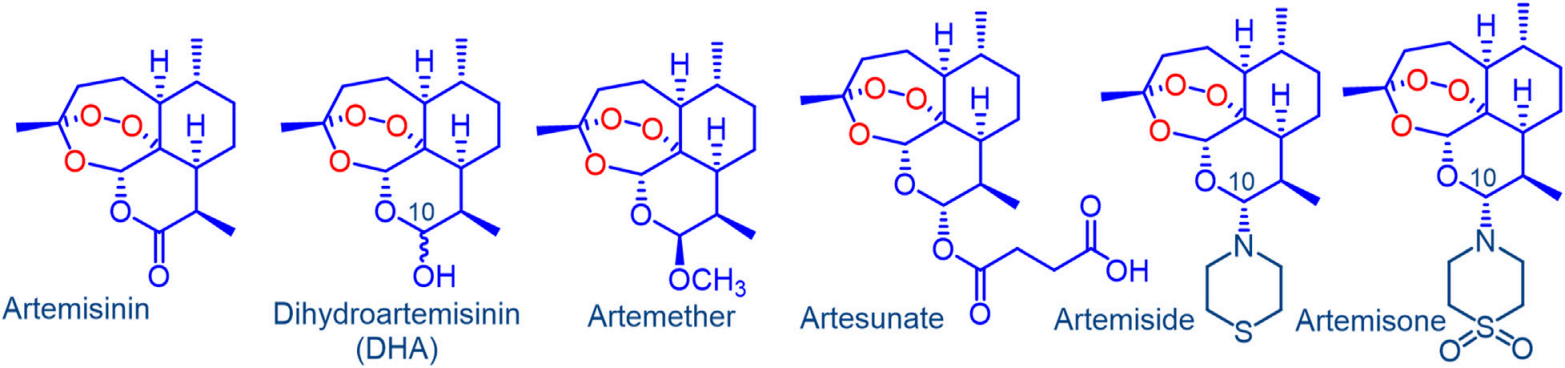
Artemisinin, clinical derivatives dihydroartemisinin (DHA), artemether, artesunate and the amino-artemisinins artemiside and artemisone. Artemether and artesunate are rapidly converted into DHA *in vivo* via facile metabolism or hydrolysis respectively. As a hemiacetal, DHA is intrinsically unstable, and rearranges irreversibly under physiological conditions into a peroxyhemiacetal that in turn rearranges to the inert deoxyartemisinin. In contrast, the amino-artemisinins do not provide DHA on metabolism.

The discovery of artemisinin and development of its derivatives dihydroartemisinin (DHA), artemether and artesunate (Figure 1) during the latter part of the 20th Century (Jianfang et al., 2005; Cui and Su, 2009; Li et al., 2017) and their adoption on a world-wide basis effectively served to counter the threat posed by resistance of the malaria parasite, principally *Plasmodium falciparum* (*Pf*), to the hitherto most widely used drug chloroquine (CQ). Fixed-dose combinations of the artemisinins with longer half-life drugs in combination therapies (ACTs) were subsequently used with considerable success (Cui and Su, 2009; Angus, 2014). Their widespread deployment coupled with rapid parasite clearance times induced by the artemisinin component and high cure rates led to renewed optimism of world-wide eradication of malaria. However, during the first decade of the 21st Century, it became evident that the parasite was evolving to become more tolerant to artemisinin therapy—thus, longer parasite clearance times in patients treated with ACTs became increasingly apparent; the phenomenon was originally noted in Cambodia from where CQ resistance originated (Rogers et al., 2009; Phyo et al., 2016; Hamilton et al., 2019). More recently, formal resistance of the parasite to the longer half-life partner drugs in the ACT including piperaquine and mefloquine has emerged, resulting in treatment failures with ACTs (Ouji et al., 2018; World Health Organization, 2018). The problem now is manifest in India and Africa (Hanboonkunupakarn et al., 2022). Thus, yet again, after the chloroquine debacle (Majori, 1999; Greenwood et al., 2008), the goal of achieving global malaria eradication has been curtailed, and in 2019, focus turned to reducing malaria deaths by at least 90% by 2030 over those reported in 2015 (World Health Organization, 2018; World Health Organization, 2019).

As an immediate response to countering the artemisinin tolerance, or “partial resistance” (Wang et al., 2019a; Duffy and Avery, 2022), treatment regimens with a single ACT have been prolonged, two different ACTs have been administered in sequence (Tun et al., 2018; Hanboonkunupakarn et al., 2022; Whalen et al., 2023), and regimens of an ACT in combination with an established third antimalarial drug that in effect comprise an artemisinin triple combination (TACT) have been used (van der Pluijm et al., 2020; Hanboonkunupakarn et al., 2022). These temporal modifications have been criticized (Wang et al., 2019b; van der Pluijm et al., 2020; Xu et al., 2022), as clearly, successful long-term deployment of TACTs require that these be markedly superior to current ACTs. In order to drive compliance, their use must be supported officially by the WHO and international funders (Bolarinwa et al., 2023). Exposure of the non-artemisinin drug of currently used ACTs and TACTs will elicit formal resistance to that drug given the extent of parasite knockdown by the artemisinin is now insufficient to protect the non-artemisinin component (Wang et al., 2019a). Careful analyses of the problem, the nature of the propagation of the resistant parasites, and an appraisal of how infections due to such parasite strains should be treated have also been presented: development of new TACTs must focus *inter alia* on maximizing parasite exposure to the artemisinin derivative and should include a second partner drug with a half-life matching that of the other long-acting partner drug (Erhunse and Sahal, 2021; Masserey et al., 2022; Bolarinwa et al., 2023). Yet, in spite of these careful recommendations, no regard is accorded to mechanism of action of the component drugs of the combination, much less how such mechanism of action of one individual drug will work to assist drug action of the other components.

Irrespective of this, such enhancement of parasite exposure cannot be achieved with the current artemisinin derivatives (Figure 1). These artemisinins, developed and first evaluated in China under the remarkable Project 523 in the late 1970s and early 1980’s, have been in use for over 40 years and their pharmacokinetic (PK) and pharmacodynamic (PD) properties are thoroughly established (Jianfang et al., 2005; Li et al., 2017). Artemether and artesunate are rapidly metabolized to the fraught DHA that in turn rearranges to inert end products (Haynes et al., 2007a, 2010; Jansen, 2010; Kotoni et al., 2014; Parapini et al., 2015). In view of the instability of DHA, it is surprising, and indeed disappointing, that development of yet newer formulations is underway (Medicines for Malaria Venture, 2023). The call for new, more active artemisinins has already been made clearly enough (Wang et al., 2019b). It is apparent also that these should not be metabolized to DHA. In this respect, amino-artemisinins such as artemiside and artemisone (Figure 1) (Haynes et al., 2004, 2006), and the metabolically and thermally robust 11-aza-artemisinins (Haynes et al., 2007b; Harmse et al., 2015; Watson et al., 2021) can be considered. The greatly enhanced parasiticidal activities of the amino-artemisinins against the various blood and liver stages of plasmodium parasites *in vitro* and *in vivo*, PK/PD properties featuring enhanced half-lives and lack of metabolism to DHA are discussed in detail elsewhere (Nagelschmitz et al., 2008; Coertzen et al., 2018; Wong et al., 2020; Gibhard et al., 2021; Watson et al., 2021).

**FIGURE 2:**
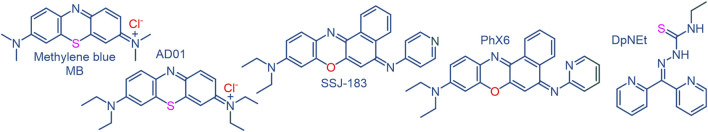
Redox active compounds methylene blue MB and the benzo [α]phenoxazine SSJ-183 each of which display synergism with artemisinins; the diethyl analogue AD01 of MB, the benzo [α]phenoxazine PhX6 and the thiosemicarbazone DpNEt are used in this study.

Although artemisone elicits uniquely equipotent activities against artemisinin-sensitive and -resistant *Pf* clones (Lanteri et al., 2014; Coertzen et al., 2018; Sissoko et al., 2020), exposure of *Pf* W2 ring stage parasites to artemisone *in vitro* according to the ring stage assay (RSA) used as a marker of artemisinin resistance does induce dormancy (Witkowski et al., 2013; Grobler et al., 2014). Resistance is not associated with binding of artemisone to the transcription factor *Pf* phosphatidylinositol-3-kinase *Pf*PI3K (Mbengue et al., 2015), but rather reflects induction of an enhanced stress response by the parasite (Tun et al., 2018; Behrens et al., 2021; Mok et al., 2021; Duffy and Avery, 2022; Egwu et al., 2022), observations that likely reflect the antimalarial mechanism of action. The artemisinin rapidly oxidizes reduced flavin cofactors of crucial intraparasitic flavoenzyme disulfide reductases and is thereby irreversibly reduced to an inert end-product. With loss of redox homeostasis, oxidative stress is increased through enhanced generation of reactive oxygen species (ROS) and concomitant modulation of stress pathways (Haynes et al., 2010, 2011, 2012; Coertzen et al., 2018). In order to ensure prolongation of oxidative stress, the oxidant artemisinin is best combined with a redox (or ‘pro-oxidant’) (Wondrak, 2009) drug such as MB or related phenothiazine (Vennerstrom et al., 1995; Joanny et al., 2012), phenoxazine (Ge et al., 2010), naphthoquinone (Sidorov et al., 2016; Ahenkorah et al., 2020) or others (Wondrak, 2009; Dharmaraja, 2017). MB, an active antimalarial drug in its own right (Akoachere et al., 2005; Adjalley et al., 2011; Dormoi et al., 2012), rapidly oxidizes reduced flavin cofactors of flavin disulfide reductases (Haynes et al., 2011, 2012) and is thereby reduced to leucomethylene blue. The latter is oxidized by oxygen to MB to generate ROS in a redox cycling process. Thus, the benefit of combining the oxidant artemisinin with the redox drug accrues through the artemisinin abruptly inducing oxidative stress that is maintained by cycling of the redox drug (Coertzen et al., 2018). Thus, additive or synergistic interactions between the drug partners obtain (Haynes et al., 2012). In this respect, the benzo [α]phenoxazine SSJ-183 (Figure 2) is potently active against blood stage asexual parasites, in particular ring-stage parasites, and displays synergism with artemisinin (Ge et al., 2010; Wittlin et al., 2013). Thiosemicarbazones such as DpNEt (Figure 2) are not intrinsically redox active, but form complexes with redox-active metal ions such as Fe(III) or Cu(II) *in situ* (Kalinowski et al., 2007; Helsel and Franz, 2015; Heffeter et al., 2019; Parkinson et al., 2019). The redox cycling proceeds via intracellular reduction of the chelated metal ion by endogenous thiols, or more efficiently by reduced flavin cofactors (Woodmansee and Imlay, 2002; Petrat et al., 2003), to Fe(II) or Cu(I) that are reoxidized by oxygen to generate ROS. The antimalarial activity of such compounds is thoroughly established (Klayman et al., 1979). The most active incorporate a pyridyl ligand for optimum chelation of the metal ion (Akladios et al., 2019; Parkinson et al., 2019). Overall then, the current study focusses on developing new drug combinations with emphasis on enhancement of efficacy by virtue of the respective mechanism of action of the component drugs.

To complete the TACT platform, a highly active third drug with a distinct mode of action incorporating activity against artemisinin-resistant parasites must be used; we have discussed elsewhere the rationale for using such drugs based on the decoquinate scaffold (Beteck et al., 2018; Watson et al., 2022).

Here, we evaluate the suitability of redox drugs to be partnered with artemisone, and eventually, with artemiside. These are the diethyl analogue AD01 of MB that with its *N*,*N*-diethylamino group is more lipophilic than MB (Delport et al., 2014), the phenoxazine PhX6, and the structurally disparate thiosemicarbazone DpNEt (Kalinowski et al., 2007). The efficacies of these compounds against *Pf*, how these are affected in combinations with artemisone, and DMPK parameters are recorded. A critical comparison of the data of these compounds is then provided, to enable selection of redox active compounds and their combinations with the amino-artemisinins for ongoing studies.

## Materials and methods

### Materials

Gentamicin and chloroquine were obtained from Sigma-Aldrich, Johannesburg, South Africa and used as received. The ethyl analogue AD01 (Delport et al., 2014) of methylene blue, the phenoxazine PhX6, the thiosemicarbazone DpNEt (Kalinowski et al., 2007; Parkinson et al., 2019) and artemisone, ≥95% purity (Chan et al., 2018; Coertzen et al., 2018; Gibhard et al., 2021), were prepared and assayed for purity as previously described (Chan et al., 2018; Gibhard et al., 2021; Watson et al., 2021) RPMI, glutamine, glucose, Hepes buffer, hypoxanthine, albumax and foetal calf serum were obtained from Celtic Molecular Diagnostics, Cape Town, South Africa. Dulbecos Modified Eagles Medium was obtained from Highveld Biologicals, Cape Town, South Africa. Acetonitrile, potassium dihydrogen phosphate and dipotassium hydrogen phosphate were purchased from Merk, Darmstadt, Germany. Analytical grade dimethyl sulfoxide (DMSO), HAMS F12 medium, formic acid, carbamazepine, hydrocortisone, propranolol hydrochloride, verapamil and vinpocetin were obtained from Sigma-Aldrich, St Louis, MO, United States. Water was purified by Millipore Elix 10 reverse osmosis and a Milli-Q (Millipore, United States). Human blood and plasma were obtained from the Western Province blood transfusion services, Cape Town, South Africa, and liver microsomes were obtained from Xenotech, Kansas City, KS, United States. All other reagents were of analytical grade.

### Efficacies against *Plasmodium falciparum* NF54 and DD2 using lactate dehydrogenase assay

Screening was carried out with the chloroquine-sensitive (CQS) *P. falciparum* NF54 and the chloroquine-resistant (CQR) DD2 strains, obtained from the Malaria Research and Reference Reagent Resource (MR4) repository. *P. falciparum* strains were continuously cultured at 37°C in human O+ erythrocytes using a modified version of the method described by Trager and Jensen (Trager and Jensen, 1976). The culture medium comprised 10.4 g/L RPMI 1640 (with glutamine and without NaHCO_3_), 4 g/L glucose, 6 g/L Hepes buffer, 0.088 g/L hypoxanthine, 5 g/L albumax, and 102 mL/L (0.05 g/L) gentamicin at pH 7.4. The half maximal inhibitory concentration (IC_50_) of each compound was determined using a modified version of the parasite lactate dehydrogenase (pLDH) assay over 72 h (Makler et al., 1993). The assay was performed in quadruplicate. The data were analyzed by nonlinear regression analysis using the GraphPad PRISM version 5.00 program to determine the IC_50_ of each compound. Chloroquine was used as reference standard.

### Cytotoxicity assays

Cytotoxicity assays were conducted against the Chinese Hamster Ovary (CHO) cell line, ATCC CCL-61, strain CHO-K1. The cells were cultured in a medium consisting of 10% foetal calf serum, 45% Dulbecos Modified Eagles Medium (DMEM) and 45% HAMS F12 medium (1:1). Cells were grown at 37°C in a humidified atmosphere of 5% CO_2_ and maintained by passage. Cells lines were seeded in 96-well microtitre plates at 10^4^ cells/well in cell medium. Once seeded, cells were incubated at 37°C for 24 h before the test samples were added. Cell viability was determined using the colorimetric 3-(4,5-dimethylthiazol-2-yl)-2,5-diphenyl tetrazolium bromide (MTT) assay (Mosmann, 1983). DMSO (100 µL) was added to dissolve the MTT formazan precipitate, and the plates were gently agitated for 2 min to ensure homogeneity. Absorbance was measured at 540 nm using a Turner BioSystems, Inc. ModulusTM II Microplate Reader. The data were analyzed by nonlinear regression analysis using the GraphPad PRISM version 5.00 program to determine the IC_50_ of each compound.

### ADME assays

i. kinetic solubility: Stock concentrations of test samples (10 mM) were prepared in DMSO and diluted to a final concentration of 200 µM in phosphate buffer saline (PBS) solutions at pH 2, 6.5, and 7.4 in flat-bottomed 96-well plates. A three-point calibration curve of each control and test compound (11, 100 and 220 µM) was prepared from stock solutions in DMSO. Plates were agitated on an orbital shaker at room temperature for 2 h at 200 rpm and subsequently analysed using a reverse phase Gemini NX-C18 column (5 μM, 2.1 mm × 50 mm) with an Agilent 1,200 Rapid Resolution HPLC with diode array detection. The aqueous mobile phase was water with 0.1% formic acid and the organic mobile phase was acetonitrile with 0.1% formic acid. The gradient method has a flow rate of 0.8 mL/min, with a total run time of 2.5 min starting at 5% organic and increasing to 95% at 1.4 min, holding for 40 s before returning to 5% to re-equilibrate the column.
ii. Lipophilicity. Stock solutions (10 mM) of compounds were diluted to 100 μM in 96-well deep-well plate containing 300 µL of 1-octanol and 300 µL of phosphate buffer at pH 7.4. The plate was agitated on an orbital shaker at 750 rpm for 2 h. The organic and buffer layers of each well were transferred separately to a new analysis plate. The samples were analysed with the HPLC diode array detection as described for kinetic solubility. Samples from both layers were analysed to determine Log_D_7.4 values.
iii. Passive permeability. A parallel artificial membrane permeation assay (PAMPA) was used to assess the passive permeability of compounds with a 96-well MultiScreen filter plate (0.4 μM pore size, Millipore). The filter plate was precoated with 5% hexadecane in hexane. Stock solutions of compounds were diluted to 1 mM in a donor buffer (pH 6.5). Lucifer yellow was added to the apical wells of the precoated MultiScreen plate containing compound donor solution. The donor buffer solutions were spiked with each test compound (1 mM). The acceptor-buffer solution was prepared by adding 10 μL DMSO to 990 μL acceptor buffer (pH 7.4) and 250 μL were added to the basolateral (acceptor) wells. The donor plate was carefully inserted into the acceptor plate and incubated at room temperature for 4 h. After the incubation period, 50 μL of samples from the acceptor plate were transferred to a round bottom 96-well plate, together with 30 μL of donor buffer for matrix matching. Theoretical equilibrium samples for each test compound were prepared by adding 150 μL of donor solutions to 250 μL acceptor solutions and left at room temperature. These theoretical equilibrium samples represent complete transfer of the compound into the donor well. For the theoretical equilibrium samples, 80 μL were added to the analysis plate. Lastly, 160 μL of the internal standard carbamazepine (0.1 μM) was added to all acceptor and equilibrium samples. A portion of the sample containing Lucifer yellow was analyzed on a BioRad iMarkTM Microplate Absorbance Reader (BioRad, Hercules, CA, United States; excitation 490 nm, emission 510–570 nm) to determine that Papp was within the acceptable range (<50 nm/s). Samples were analysed using reverse phase Gemini NX-C18 column (5 μm, 2.1 mm × 50 mm) with a Shimadzu HPLC coupled with an AB Sciex 3200 Q TRAP MS). LogP_app_ (apparent permeability) was calculated using the following equations:

$$C=\frac{V_{D\;}\times V_{A}}{\left(V_{D}+V_{A}\right)\times A\times t\;}$$ (1)

where V_A_ = volume of donor compartment (0.15 cm^3^), V_D_ = volume of acceptor compartment (0.25 cm^3^), A = accessible filter area (0.24 cm^2^) and t = incubation time (14,400 s).

$$P_{app}=C\times -\ln\;(1-\frac{\left[{drug}_{acceptor}\right]}{\left[{drug}_{equilibrium}\right]}$$ (2)

Where [drug_acceptor_] is the analyte to internal standard peak area ratio of the test compound in the acceptor compartment and [drug_equilibrium_] is the analyte to internal standard peak area ratio of the combined total donor and acceptor compartments.

iv. Plasma stability. Stock solutions of compounds in DMSO (10 mM) were used to spike pooled human plasma. 90 μL of each sample was transferred in duplicate to 6 different wells of a 96-deep well plate. An aliquot of each sample was immediately quenched with ice cold acetonitrile containing the internal standard carbamazepine (0.1 µM), after which the plate was incubated in a water bath (37°C for 1 h). Aliquots of samples were removed at 5, 15, 40, 60 and 180 min and quenched with ice cold acetonitrile. After samples of the final time point were precipitated, the supernatant was transferred to a round bottom 96-well plate for analysis by LC-MS/MS using a reverse phase Gemini NX-C18 column (5 μm, 2.1 mm × 50 mm) with a Shimadzu HPLC coupled with an AB Sciex 3200 Q TRAP MS) operated at unit resolution in the multiple reaction monitoring (MRM) mode. The precursor ions, product ions and mass spectrometer conditions are summarised in Table 1. The aqueous phase consisted of water with 0.1% formic acid and the organic mobile phase consisted of methanol and acetonitrile (1:1, (*v/v*)) with 0.1% formic acid. The flow rate was 0.5 mL/min with 60% organic for 3 min followed by an increase to 95% for 2 min before returning to 60%. The peak area ratios were used to calculate the amount of parent compound remaining and t_1/2_ in plasma, using the following equations.

**TABLE 1:** Multiple reaction monitoring transitions and final mass spectrometer conditions.

Analyte	Transition	Dwell time (ms)	Declustering potential (V)	Entrance potential (V)	Collision energy (V)	Cell exit potential (V)
PhX6	395 → 351	150	131	9	31	6
	395 → 78.2	150	131	9	77	6
AD01	341 → 297	150	61	11	47	6
	341 → 253	150	61	11	71	12
DpNEt	286 → 241	150	46	5	15	6
	286 → 183	150	46	5	12	6

Eq. 3 % Parent remaining

$$\%\;Parent=\frac{Normalised\;peak\;area\;of\;sample\;at\;timepoint}{Normalised\;peak\;area\;of\;sample\;at\;t=0}\;x\;100$$ (3)

Eq. 4 Predicted t_1/2_

$$t_{\frac{1}{2}}=-0.693/\lambda$$ (4)

Where λ is the slope of the Ln % parent remaining vs. time curve.

v. Plasma protein binding. Plasma protein binding was determined in pooled human plasma using an ultracentrifugation method. Stock solutions of test compounds (10 mM) were diluted in phosphate buffer and spiked into plasma in deep well 96-well plates (10 µM). An aliquot of each compound was immediately precipitated with ice cold acetonitrile containing the internal standard carbamazepine (0.1 μM). These served as total concentration samples. After incubation in a water bath at 37°C for 1 h, the samples were transferred to ultracentrifuge tubes in duplicate and centrifuged for 4 h at 37°C and 30,000 g (Optima L-80XP, Beckman). Following centrifugation, the supernatant was transferred to the plate containing the total concentration samples and precipitated with ice cold acetonitrile containing the internal standard. Analyte concentrations of all compounds were determined via LC-MS/MS (reverse phase Gemini NX-C18 column and Shimadzu HPLC coupled with an AB Sciex 3200 Q TRAP MS) as described above.
vi. Metabolic stability. A 5-point metabolic stability assay in human and mouse liver microsomes were performed in duplicate in 96-well plates. Stock solutions of AD01, PhX6 and DpNEt were prepared in DMSO (0.4 mM) and were then individually incubated at 37°C in mouse and human liver microsomes (0.4 mg/mL). An aliquot was removed at 0 min and quenched with ice cold acetonitrile containing the internal standard carbamazepine (0.1 μM). The cofactor NADPH was added, and the reactions were subsequently stopped at time points 5, 10, 30 and 60 min by removal of an aliquot and quenching with ice cold acetonitrile containing the internal standard. The supernatant of all samples was analysed via LC-MS/MS analysis (reverse phase Gemini NX-C18 column and Shimadzu HPLC and AB Sciex 3200 Q TRAP MS) as described above. Calculation of *in vitro* t_1/2_, intrinsic clearance rate (CL_int_) and predicted *in vivo* clearance (CL_H_) was carried out using Eqs 3, 4 as shown above, and Eq. 5 (below).

Eq. 5 CL_int_

$${Cl}_{\mathrm{int}}=\frac{0.693}{t_{\frac{1}{2}}\;\left(\min\right)}\times \frac{Volume\;of\;incubation\;\left(\mu L\right)}{microsomal\;protein\;\left(\mu g\right)}$$

Where t_1/2_ is calculated in minutes.

### *In vivo* PK studies

i. Animals. Healthy C57BL/6 mice each weighing approximately 25 g were maintained at the University of Cape Town animal facility. Mice were housed in 27 × 21 × 28 cm cages (n = 3) under controlled environmental conditions at 22°C ± 2°C, humidity of 55% ± 15% and a 12-h light/dark cycle. Food and water were available *ad libitum.* Mice were acclimatised to the experimental environment for 4–5 days prior to initiating the experiments.
ii. Oral drug administration. AD01 and PhX6 were prepared in 100% Millipore water while DpNEt was prepared in an aqueous suspension of 0.5% HPMC (*w/v*) containing 0.2% Tween 80 (*v/v*). The weighed compounds were sonicated and vortexed to ensure all solutions were homogenous. Oral dosing of 4.6 mg/kg, 4.5 mg/kg and 5 mg/kg was achieved via oral gavage for AD01, PhX6 and DpNEt respectively. The total volume per administration was 200 μL. Blood samples were collected post-dose in lithium heparin microvials via tail bleeding at predetermined intervals at 0.5, 1, 3, 5, 8, 10 and 24 h. Samples were gently vortexed and stored at −80°C until analysis.
iii. Intravenous administration. AD01 and PhX6 were prepared in 100% Millipore water while DpNEt was prepared in an organic vehicle consisting of 10% *N*,*N*-dimethylacetamide, 30% polyethylene glycol 400 (PEG), 50% polypropylene glycol (PPG) and 10% ethanol (1:3:5:1, v/v) for intravenous (*iv*) dosing at 10 mg/kg. The total volume of administration was 80 μL. Blood samples were collected post-dose in lithium heparin microvials via tail bleeding at predetermined intervals at 0.16, 0.5, 1, 3, 5, 8, 10 and 24 h. Samples were gently vortexed and stored at −80°C until analysis.
iv. Sample processing and analysis. A quantitative LC-MS/MS assay was used to determine whole-blood concentrations of AD01, PhX6 and DpNEt. Whole blood samples (20 μL) were treated with 100 μL ice cold acetonitrile containing the compound RMB073 used previously for determination of whole blood concentrations in analysis of TB-active compounds, as internal standard (200 ng/mL) (Watson et al., 2022). The mixture was vortexed vigorously for 1 min, and 5 µL of the supernatant were injected onto the analytical column following centrifugation (5590 g for 5 min). A Shimadzu HPLC and reverse phase Gemini NX-C18 column coupled to an AB Sciex 3200 Q TRAP MS was operated at unit resolution in the multiple reaction monitoring (MRM) mode as described above. The calibration standards ranged from 7.80 to 4,000 ng/mL for AD01, 3.9–4,000 ng/mL for PhX6 and 15.6–4,000 ng/mL for DpNEt.
v. Data analysis. Drug concentration versus time plots for each compound were used to determine maximal drug concentration *C*_max_, time *T*_max_ to reach *C*_max_, elimination half-life *t*_1/2_ and the area under the concentration-time curve from time zero to infinity, AUC_0-inf_. From these values the PK parameters clearance CL, volume of distribution Vd and oral bioavailability BA were determined using the non-compartmental analysis Microsoft Excel Add-In PKSolver (Zhang et al., 2010).

### Fixed ratio combination experiments

Artemisone was used in the combination studies with each of AD01 and PhX6. Combination studies were conducted using a modified version of the method described by Fivelman et al. (2004) (Fivelman et al., 2004). Against drug-sensitive NF54 *P. falciparum* parasites in the standard 72-h pLDH assay. The drug stocks were then mixed to make 6 different volumetric ratios to obtain a range of concentrations (Table 2). Concentration ranges for combinations were prepared from the predetermined single IC_50_ values (Table 3) such that the IC_50_ of the individual compounds would be in the middle of the plate. The FIC_50_ and ∑FIC were calculated as described in Eqs 6–8 (Fivelman et al., 2004).

**TABLE 2:** Fixed ratio combinations and concentration of each drug in each combination.

Combinations	Ratio	Concentration (nM)		
		Compound A	Compound B	
		Artemisone	AD01	PhX6
C1	1:0	100	0	0
C2	3:1	75	25	200
C3	1:1	50	50	400
C4	1:3	25	75	600
C5	1:9	10	90	720
C6	0:1	0	100	800

**TABLE 3:** Mean antimalarial activities and cytotoxicities for AD01, PhX6, DpNEt and artemisone *in vitro* with associated controls, *n* = 3.

Compound	*Plasmodium falciparum* IC_50_ (nM)		Chinese hamster ovary IC_50_ (µM)	Resistance index^a^	Selectivity index^+^	
	NF54	DD2			CHO/sensitive *Pf* strain	CHO/resistant *Pf* strain
AD01	23.1 ± 2.5	11.9 ± 2.8	74 ± 9.4	0.5	>1,000	>1,000
PhX6	41.8 ± 0.04	16.2 ± 2.4	192 ± 4.6	0.4	>1,000	>1,000
DpNEt	40.7 ± 1.4	28.6 ± 4.0	11 ± 4.2	0.7	270	384
AMS	4.50 ± 2.42	5.48 ± 2.03	>249 ± ND	1.2	>1,000	>1,000
CQ	9.9 ± 0.4	142 ± 16.8	ND	14.3	ND	ND

^a^ Resistance index = IC_50_ (resistant strain)/IC_50_ (sensitive strain), + Selectivity Index = cytotoxicity IC_50_/activity IC_50_; ND, not determined; CHO, chinese hamster ovary; AMS, artemisone; CQ, chloroquine.

Eq. 6: FIC_50_ of compound A

$${FIC}_{50}\left(A\right)=\frac{{IC}_{50}\;\left(A+B\right)}{{IC}_{50}\;A}$$

Eq. 7: FIC_50_ of compound B

$${FIC}_{50}\left(B\right)=\frac{{IC}_{50}\;\left(B+A\right)}{{IC}_{50}\;B}$$

Eq. 8: Sum of FIC_50_

$$\sum {FIC}_{50}={FIC}_{50\;\left(A\right)}+{FIC}_{50\;\left(B\right)}$$

Finally, FIC_50_ values of compounds in respective combinations were plotted as isobolograms.

## Results

### Biological activities

Antimalarial activities *in vitro* (<50 nM) against both drug sensitive (NF54) and chloroquine resistant DD2 *P. falciparum* strains are given in Table 3. In accord with previous data, artemisone possesses potent antimalarial activity against *Pf* drug sensitive and resistant parasite strains (Wong et al., 2020). SSJ-183, the lead benzo [α]phenoxazine closely related to PhX6 (Figure 2), possesses activity according to the tritiated hypoxanthine assay against the multidrug resistant *Pf* K1 strain of 7.6 nM, suggesting it is more potent than PhX6; it also possesses low nM IC_50_s against other *Pf* strains, highlighting the potential of this drug class (Wittlin et al., 2013). IC_50_ activities for DpNEt against CQ-sensitive *Pf* NF54 and CQ-resistant Dd2 strains are noted to be 40.7 and 28.6 nM respectively (Table 3); values obtained previously according to the lactate dehydrogenase assays used here are 14.1 and 16.8 nM (Woodmansee and Imlay, 2002). DpNEt was the only compound which displayed cytotoxicity against the CHO cell line. However, its selectivity index is within the acceptable range (Pink et al., 2005). None of the compounds evaluated exhibited cross resistance with chloroquine (Resistance Index <1, Table 3). Consequently, all three compounds were taken forwards to pharmacokinetic evaluation and combination studies.

### ADME

The ADME properties of the three redox active compounds are summarised in Table 4. The MB analogue AD01 and the thiosemicarbazone DpNEt are predicted to be soluble (>150 μM) at all pH levels. The phenoxazine PhX6 has low predicted solubility at pH 6.5 and 7.4 (<5 μM), and high predicted solubility at pH 2 (>150 μM). This is likely due to the compound existing predominantly as the protonated conjugate acid at lower pH. AD01 has low lipophilicity (LogD_7.4_ 0.2). In comparison, MB has a log *p*-value of −0.9 at pH 7, and its log D value at 0.06 is lower than that of AD01 (Xing and Glen, 2002). Notably, the lipophilicity of PhX6 (LogD_7.4_ 1.0) is at the lower end of the preferred range while that of DpNEt (LogD_7.4_ 2.3) is classified as ‘ideal’ (Haynes et al., 2006; Bampidis et al., 2019).

**TABLE 4:** *In vitro* kinetic solubility, microsomal and plasma stabilities, and plasma protein binding of AD01, PhX6 and DpNEt, *n* = 3.

Compound	Predicted solubility µM			Lipophilicity LogD_7.4_	Permeability LogP_app_	Cl_int_ mL/min/kg		Plasma half-life min	Plasma protein binding %, (f_u_)
	pH 2	pH 6.5	pH 7.4			Mouse	Human		
AD01	>150	>150	>150	0.2	−6.4	80.6	16.2	85	ND*
PhX6	>150	<5	<5	1.0	−4.4	88.2	20.2	>150	90 (0.10)
DpNEt	>150	>150	>150	2.3	−3.7	22.3	4.0	>150	94 (0.06)

ND* = not determined due to plasma instability; Cl_int_, intrinsic liver clearance rate; f_u_ fraction unbound.

High apparent permeability was observed for PhX6 (LogP_app_ −4.4) and DpNEt (LogP_app_ −3.7), suggesting that passive diffusion likely contributes significantly to the absorption of these compounds; indeed, the extent of absorption is predicted to be high (Kansy et al., 1998). It should be noted that PhX6 is likely to be poorly soluble at pH 6.5 which may have resulted in the apparently lower rate of passive diffusion compared to DpNEt, although the differences in lipophilicity values could also be a driver. The high permeability of DpNEt which will allow access to labile iron reservoirs may be linked to the good biological activity (Richardson et al., 2006). AD01 (LogP_app_ −6.4) expressed moderate to poor apparent permeability, indicating the quantity of compound that was able to cross from the apical to the basolateral side of the membrane was limited. The lower permeability is in line with the behaviour of amphipathic substrates and has also been observed with MB (Agency, 2009). Notably, AD01 had low stability in plasma with only 21% remaining after 3 h and this prevented determination of its plasma protein binding. This plasma stability is significantly lower than that of MB which was found to be 94% protein bound (Agency, 2009). PhX6 and DpNEt had good stability and >150 min half-life in plasma and were found to be highly protein bound. AD01 had high maximum predicted hepatic clearance (Cl_int_) in both species (80.6 mL/min/kg and 16.2 mL/min/kg for mouse and human respectively, Table 4). This may be due to rapid metabolism, but the data also suggests general lability in the biological medium. Predicted CL_int_ for PhX6 was similarly high for both mouse (88.2 mL/min/kg) and human (20.2 mL/min/kg) species (Li et al., 2003). DpNEt had a very low predicted human hepatic clearance rate (4.00 mL/min/kg).

### Pharmacokinetics *in vivo*

**FIGURE 3:**
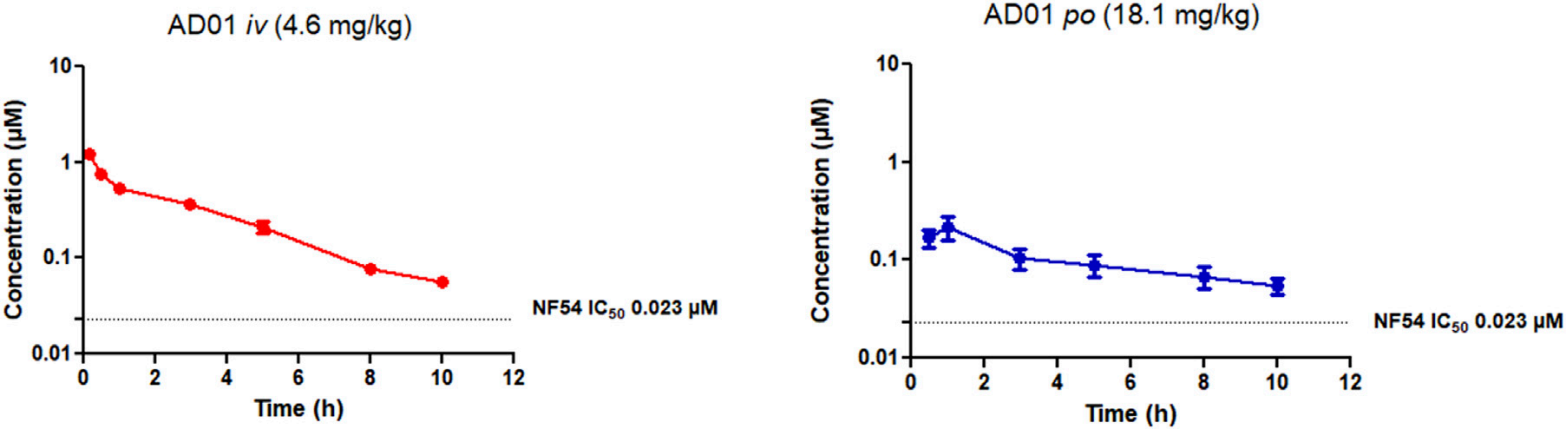
Mean ± SEM concentration-time profiles of AD01 following intravenous (*iv*) and oral (*po*) administration. The dotted line represents IC_50_ activity of PhX6 against *P. falciparum* NF54.

**FIGURE 4:**
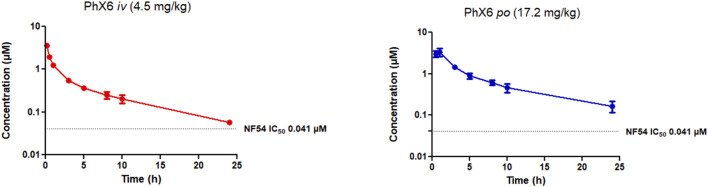
Mean ± SEM concentration-time profiles of PhX6 following intravenous (*iv*) and oral (*po*) administration. The dotted line represents IC_50_ activity of PhX6 against *P. falciparum* NF54.

**FIGURE 5:**
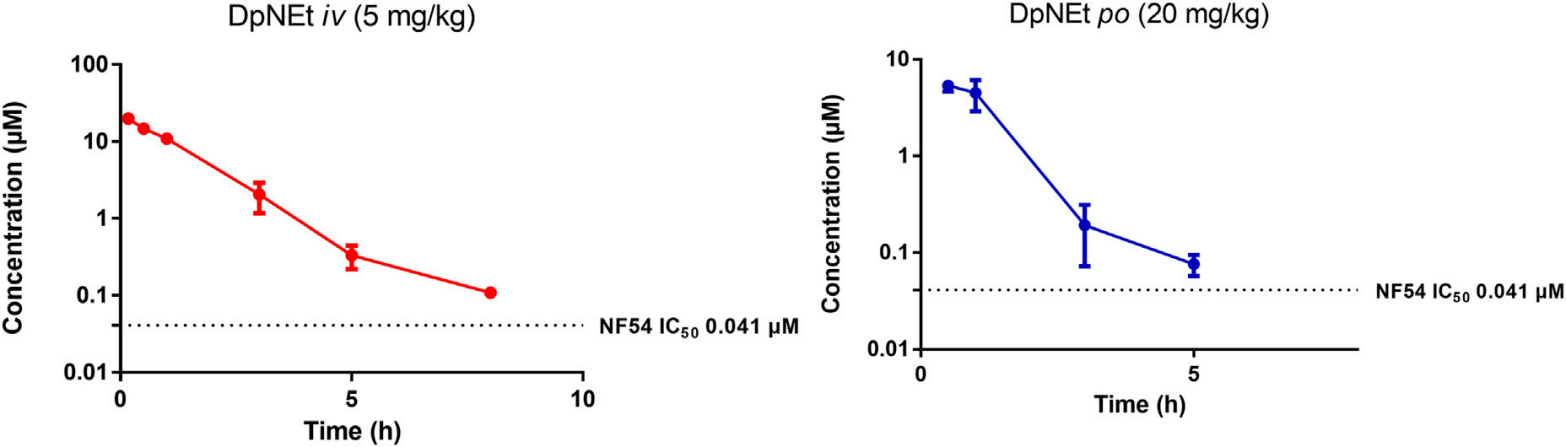
Mean ± SEM concentration-time profiles of DpNEt following intravenous (*iv*) and oral (*po*) administration. The dotted line represents IC_50_ activity of DpNEt against *P. falciparum* NF54.

The PK parameters and the whole blood concentration profiles obtained from *iv* (*n* = 5) and *po* (*n* = 5) dosing groups in the murine model (Figures 3–5) are summarised in Table 5. AD01 was rapidly cleared following dosing with a predicted clearance rate of 74.41 ± 6.68 mL/min/kg and a low bioavailability of 15%, and short half-life of 2.51 ± 0.07 h using a single compartment model. This correlates well with the *in vitro* ADME results. While an initial distribution phase can be seen in the data, distribution is rapid, the observed distribution being complete within the first hour. Upon oral administration, AD01 reached a C_max_ of 0.25 ± 0.05 µM after 0.88 h, this value being some 10-fold higher than the IC_50_ value. The limited exposure and short half-life of AD01 is likely due to the combined effects of the rapid clearance rate seen *in vitro* and *in vivo* and observed plasma instability *in vitro*. The low bioavailability of AD01 could be due to its low lipophilicity which would limit absorption into the gastrointestinal tract. MB has been noted to be affected by efflux pumps such as P-gp and multidrug resistance proteins. Due to the close structural similarity between MB and AD01, it is likely AD01 is removed by the same mechanisms which limits exposure (Senarathna et al., 2016). There appears to be no data available on the pharmacokinetics of MB in mice, but in humans the mean terminal half-life of MB is estimated to be 5.25 h (Peter et al., 2000) and the bioavailability is 72.3± 23.9% (Walter-Sack et al., 2009). Despite the differences in the two models, the evidence indicates that AD01 does not have PK parameters superior to those of MB.

**TABLE 5:** Pharmacokinetic parameters following intravenous and oral administration in mice for each of AD01, PhX6 and DpNEt.

Compound	t_1/2_ h	T_max_ h	C_max_ µM	Vd L/kg	CL mL/min/kg	AUC_0-∞_ min∙µmol/L	BA %
Intravenous							
AD01	2.51 ± 0.07	-	-	16.24 ± 1.73	74.41 ± 6.68	186 ± 15	-
PhX6	7.96 ± 0.73	-	-	14.92 ± 2.09	21.47 ± 1.76	540 ± 44	-
DpNEt	1.12 ± 0.13	-	-	1.00 ± 0.16	10.20 ± 0.93	1795 ± 198	-
Oral							
AD01	-	0.88 ± 0.13	0.25 ± 0.05	-	-	101 ± 19	15 ± 4
PhX6							
	-	0.80 ± 0.12	3.45 ± 0.68	-	-	1,150 ± 216	60 ± 12
DpNEt	-	0.70 ± 0.12	6.32 ± 1.15	-	-	550 ± 148	8 ± 2

PhX6 displayed a favourable pharmacokinetic profile - it was cleared slowly with a predicted clearance rate of 21.47 ± 1.76 mL/min/kg, a moderate bioavailability of 60% and a half-life of 7.96 h. It is notable that the *in vivo* clearance rate varies substantially from the predicted hepatic clearance of 88.2 mL/min/kg from the *in vitro* ADME study, whereas the two values show excellent agreement in the case of AD01 (Table 4). However, the *in vivo* concentration in plasma correlation over time data (Figure 4) does show a clear two-compartment behaviour with distinct distribution to peripheral tissue and terminal elimination sections with a distribution half-life of 26 min. It is likely that the extended clearance times and resultant improvement of half-life observed *in vivo* is attributable to this distribution of PhX6 to the peripheral compartment. We propose that PhX6 is distributed to poorly perfused tissues which results in a relatively low redistribution rate from the poorly perfused tissue into the central compartment. This, in turn, acts as a residual source of PhX6 that is protected from hepatic metabolism with a sustained release of PhX6 into the bloodstream during the redistribution process. This limitation in effective delivery of PhX6 to the liver offsets the relatively high predicted hepatic clearance. Following oral administration, PhX6 reached a C_max_ of 3.45 ± 0.68 µM after 0.8 h, this being some 100-fold higher than the IC_50_ value and remaining well above the IC_50_ value for the entire 24 h of the study. Thereafter, the concentration declined as a multiphasic elimination curve. PhX6 displays similar properties to that of SSJ-183 which has a slightly longer half-life of 10.3 h and T_max_ of 1 h (Wittlin et al., 2013).

DpNEt showed a calculated clearance rate of 10.20 mL/min/kg and a high C_max_ of 6.32 µM with a very short half-life of 1.2 h and bioavailability of 8%. The *in vivo* clearance was deemed to be complete after 5 h in this study, the analysed concentration being close to the limit of quantitation. This may be attributable to the much narrower volume of distribution for DpNEt which results in a greater proportion of DpNEt reaching the liver and being subjected to hepatic extraction per unit time, offsetting the predicted lower metabolic liability compared to either AD01 or PhX6 from the earlier *in vitro* study (Table 4). Although DpNEt showed good solubility across a wide pH range, suitable lipophilicity, low clearance rate and no plasma instability *in vitro*, the expectations of superior bioavailability and exposure were not realized. Without further study it is difficult to determine the exact reasons for this poor profile. It is possible that the liver is not the main route of clearance for DpNEt, and alternative routes such as hepatobillary or renal excretion may be operating to effectively eliminate the thiosemicarbazone, thus resulting in its low bioavailability and short half-life (Smith et al., 2019). Previous studies on related compounds have indicated that thiosemicarbazones of this type are good substrates for efflux proteins such as PgP and this would be a limiting factor for bioavailability (Al-Akra et al., 2018).

### Combination studies

As the aim is to determine the best partners for future TACT regimens, drug combination studies were undertaken with AD01 and PhX6 in combination with artemisone. DpNEt was excluded due to its poor pharmacokinetic profile.

Although there is currently no standardised method to determine drug combination effects, many models are available (Wagner and Ulrich-Merzenich, 2009; Matthews et al., 2017). The fixed ratio isobologram method has been widely used in many fields including antimalarial drug discovery and development (Fivelman et al., 2004; Wagner and Ulrich-Merzenich, 2009; Matthews et al., 2017). This method is based on the Loewe additivity model and enables calculation of the fractional IC_50_ (FIC_50_) for each drug combined in a series of volumetric fixed ratios. The summation of the FIC_50_s (ΣFIC) at each ratio is used to determine the nature of the interaction between the tested compounds. A ΣFIC less than 0.8 indicates synergy, while an ΣFIC greater than 1.4 indicates antagonism. The relation is additive if the ΣFIC is equal to 0.8–1.4. The relationship can also be determined by plotting the FIC_50_ of each compound on separate axes to create an isobologram. If the trend lines of the plots have a concave shape it is considered synergistic, while a convex shape suggests antagonism (van Vuuren and Viljoen, 2011). A straight line represents additivity.

**FIGURE 6:**
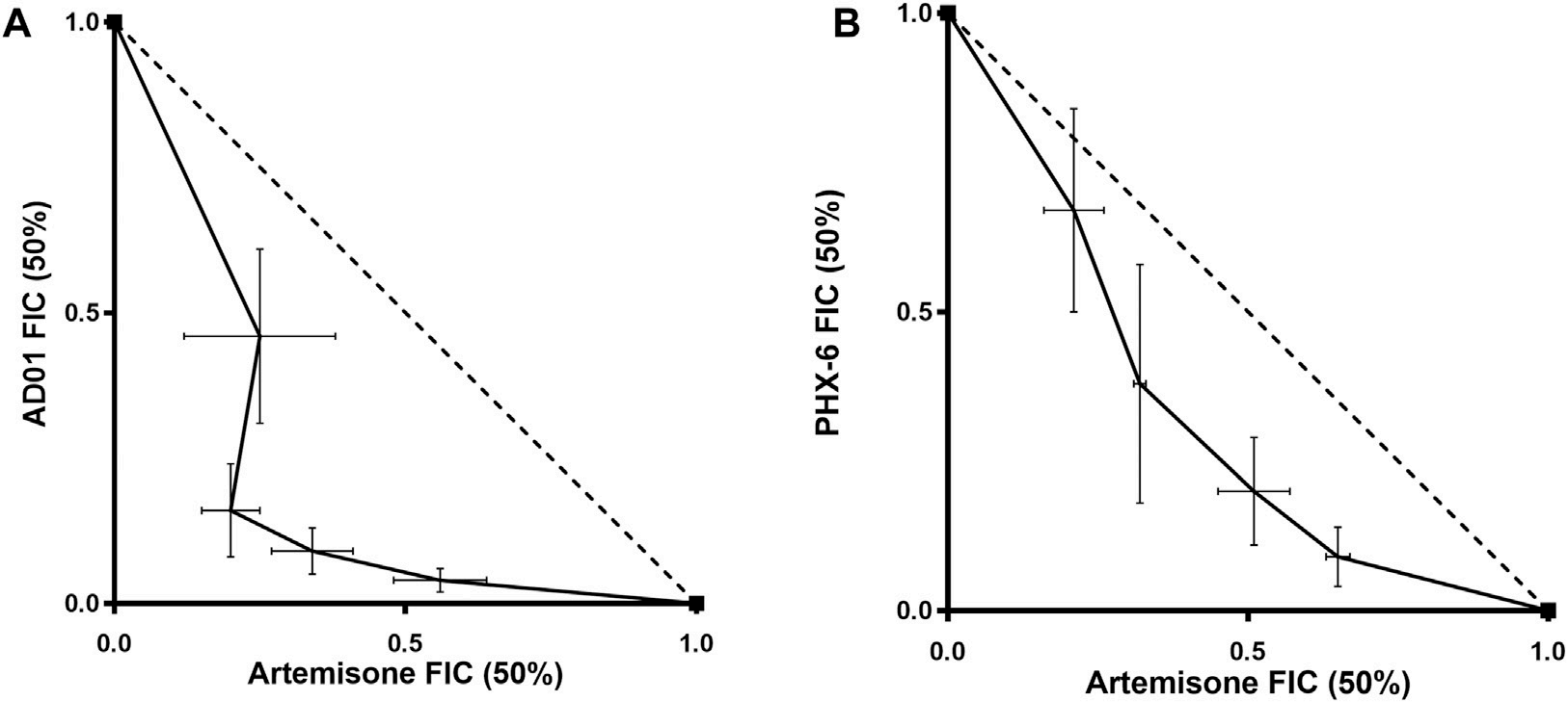
Isobolograms of fixed ratio combination experiments for artemisone and AD01 **(A)** and artemisone and PhX6 **(B)** against the CQ-sensitive *Pf* NF54 strain. The trend line of the FIC_50_s (solid line) is concave and below the additivity line (dotted line) revealing a synergistic relationship between both drugs and artemisone. Data are the mean of 3 independent experiments.

Efficacies of both AD01 and PhX6 against the CQ-sensitive *Pf* NF54 strain were enhanced synergistically when dosed in combination with artemisone as seen in Table 6 and Figure 6. Both isobolograms show a concave trend line below the additivity line and low ΣFIC values. Artemisone + AD01 displayed particularly strong synergy at each ratio especially at 1:3. Previous studies have shown that the gametocytocidal activity of artemisone is synergistically enhanced when combined with MB (Coertzen et al., 2018). However, only an additive effect was displayed for the combination against the asexual blood stage of *P. falciparum*. This suggests that AD01 may be a better partner drug than MB for the TACTs in terms of biological activity. In comparison to AD01, artemisone + PhX6 showed weaker synergy; however the combination was largely synergistic with low calculated FIC_50_’s, with the exception of the combination of 1:9, which was found to be additive.

**TABLE 6:** Mean IC_50_ and FIC_50_ of AD01+artemisone and PhX6+artemisone against CQ-sensitive *Plasmodium falciparum* NF54 strain using the fixed ratio isobologram method.

Combination	Combination ratio (A:B)	^a^Mean IC_50_ ± SD (nM)		^a^Mean FIC_50_		∑FIC_50_	Interaction
		Drug A	Drug B	Drug A	Drug B		
AMS + AD01	1:0	6.16 ± 2.92	nd	1.00 ± 0.0	nd	1.00	
	3:1	3.40 ± 1.44	0.92 ± 0.40	0.56 ± 0.08	0.04 ± 0.02	0.60	Synergy
	1:1	1.97 ± 0.83	2.04 ± 0.78	0.34 ± 0.07	0.09 ± 0.04	0.43	Synergy
	1:3	1.14 ± 0.38	3.63 ± 1.53	0.20 ± 0.05	0.16 ± 0.08	0.36	Synergy
	1:9	1.35 ± 0.54	10.35 ± 2.40	0.25 ± 0.13	0.46 ± 0.15	0.71	Synergy
	0:1	nd	23.31 ± 2.49	nd	1.00 ± 0.0	1.00	
AMS + PhX6	1:0	2.86 ± 1.01	nd	1.00 ± 0.0	nd	1.00	
	3:1	1.84 ± 0.59	3.20 ± 1.34	0.65 ± 0.02	0.09 ± 0.05	0.73	Synergy
	1:1	1.46 ± 0.46	7.88 ± 3.67	0.51 ± 0.06	0.20 ± 0.09	0.72	Synergy
	1:3	0.91 ± 0.31	14.17 ± 6.13	0.32 ± 0.01	0.38 ± 0.20	0.70	Synergy
	1:9	0.61 ± 0.35	21.21 ± 2.22	0.21 ± 0.05	0.67 ± 0.17	0.87	Addition
	0:1	nd	45.61 ± 5.69	nd	1.00 ± 0.0	1.00	

^a^ Results shown are the mean IC_50_ values and SD, from at least three independent experiments.

## Discussion

Three potential redox partners for the amino-artemisinin artemisone were evaluated for *in vitro* antimalarial activity against drug sensitive and resistant strains, *in vitro* cytotoxicity, *in vitro* ADME properties and *in vivo* pharmacokinetics in mice. Following this, suitable candidates were selected for combination studies to determine effects with artemisone. Unfortunately, the thiosemicarbazone DpNEt has a surprisingly short half-life and low bioavailability, therefore discouraging further evaluation of this compound. The MB analogue AD01 is potently active against asexual *P. falciparum* parasites and showed synergy in combination with artemisone, thus surpassing the additivity of MB with artemisone, as previously recorded (Coertzen et al., 2018). However, it displayed plasma instability and was rapidly cleared *in vivo*, although it does have an exploitable bioavailability and acceptable half-life. Ultimately, it is the benzo [α]phenoxazine PhX6 that appears to be the best partner among the compounds examined here. PhX6 showed potent antimalarial activity and host selectivity with a slow clearance rate, good bioavailability, and long half-life *in vivo*. Whilst the artemisone-PhX6 combination was not as potent as the artemisone-AD01 combination *in vitro*, PhX6 displays a synergistic relationship with artemisone, making it a suitable redox partner.

It is reported that the lead benzo[α]phenoxazine SSJ-183 (Figure 2) displays synergism in combination with artemisinin *in vitro*, possesses a desirable pharmacokinetic profile, potent *in vivo* efficacies and an acceptable toxicity profile (Ge et al., 2010; Wittlin et al., 2013). Therefore, future work will focus on evaluating the efficacies *in vivo* of the benzo [α]phenoxazines PhX6 and SSJ-183 as redox partners in combination with artemisone. Both compounds have established safety profiles, which will facilitate transitioning into human studies. Combination studies will also be carried out for artemiside, the sulfide precursor of artemisone (Figure 2) that is rapidly metabolized to artemisone and other active metabolites (Gibhard et al., 2021). With a C_max_ 2.6-fold higher than that attained following oral administration of artemisone in a murine model, coupled with a favourable toxicity profile, it is necessary to evaluate the combination of artemiside with the benzo [α]phenoxazines. Studies also directed towards the identification of the third drug to complete the proposed triple combination therapy are also ongoing (Watson et al., 2022).

The selection of drug partners in a combination therapy in general may be based on the pairing of agents that impact on different targets, differ in rate of action and persistence or both. In the current study, we have demonstrated the concept of synergy in our drive to establish new combination therapies for treatment of multidrug resistant malaria. The concept of synergy implies that, rather than simply magnifying a drug effect by utilizing a different mode of action, the partner drug can also magnify the mode of interaction of the primary drug. In this case, the action of the oxidant drug (artemisone) is directly promoted through continued disruption of the parasitic redox state elicited by the redox partner. The potential for disruption of flavin cofactor mediated futile consumption of the terminal cellular reducing agent NAD(P)H is then likely to result in rapid exhaustion of antioxidant defences such as glutathione resulting in parasite death through oxidative stress (Haynes et al., 2010, 2011, 2012; Coertzen et al., 2018). We propose that this chemical reinforcement of drug action is a powerful driver of the drug synergy discussed in the current study.

Thus to conclude, given the urgency of the need to counter the spread of artemisinin-resistant malaria, the development of *rational* TACTs for its treatment must be facilitated as rapidly as possible. The results of the current and foregoing studies (Coertzen et al., 2018; Watson et al., 2021) enable rational selection of partner drugs based on respective mechanisms of action that result in synergism, and on the all-important pharmacokinetic profile that will enable optimal exposure of the parasite to the drug partners. It is well noted that such perspectives should also characterize development of drug combinations for other targets, for example, for treatment of tuberculosis (TB) (Alffenaar et al., 2022), given especially the failure of clinical trials against TB employing drug combinations (Harris, 2023). Given further the notably intractable nature of the principle TB pathogen *Mycobacterium tuberculosis* (*Mtb*), the relationship between mechanism of action, drug exposure, efficacy, and especially any potential for acquisition of drug resistance must be established in any study leading to selection of eventual drug combinations. In this respect, we have already demonstrated that combining the oxidant artemisinin with a redox active phenoxazine related to the phenoxazine PhX6 described here results in enhanced activity against *Mtb*; indeed synergism is displayed by these drugs within an infected macrophage model of *Mtb* (Tanner et al., 2021). Thus, the validity of the methodology for development of new drug combinations for treatment of artemisinin-resistant malaria is reinforced. The unfortunate perseverance with the current ACTs risks not just a repeat of the CQ debacle involving a single drug, but carries the added risk now of rendering impotent a cluster of drugs comprising both the current clinical artemisinins and partner drugs including piperaquine and mefloquine. As noted in the Introduction, formal resistance to these drugs as used in current ACTs has already been reported.

## Data Availability

The raw data supporting the conclusion of this article will be made available by the authors, without undue reservation.
